# What Causes Eye Pain?

**DOI:** 10.1007/s40135-015-0073-9

**Published:** 2015-04-21

**Authors:** Carlos Belmonte, M. Carmen Acosta, Jesus Merayo-Lloves, Juana Gallar

**Affiliations:** Instituto de Neurociencias, Universidad Miguel Hernández-CSIC San Juan de Alicante, Avenida de la Universidad, s/n, 03202 Alicante, Spain; Instituto Fernandez Vega, Fundación de Investigación Oftalmológica, Av Doctores Fernández Vega, 34, 33012 Oviedo, Asturias Spain

**Keywords:** Eye pain, Physiological or nociceptive pain, Neuropathic pain, Transduction mechanisms, Pathobiological modulation, Nerve injury, Peripheral pain mechanisms, Dry eye, Eye inflammation

## Abstract

Eye pain is an unpleasant sensory and emotional experience including sensory-discriminative, emotional, cognitive, and behavioral components and supported by distinct, interconnected peripheral and central nervous system elements. Normal or physiological pain results of the stimulation by noxious stimuli of sensory axons of trigeminal ganglion (TG) neurons innervating the eye. These are functionally heterogeneous. Mechano-nociceptors are only excited by noxious mechanical forces. Polymodal nociceptors also respond to heat, exogenous irritants, and endogenous inflammatory mediators, whereas cold thermoreceptors detect moderate temperature changes. Their distinct sensitivity to stimulating forces is determined by the expression of specific classes of ion channels: Piezo2 for mechanical forces, TRPV1 and TRPA1 for heat and chemical agents, and TRPM8 for cold. Pricking pain is evoked by mechano-nociceptors, while polymodal nociceptors are responsible of burning and stinging eye pain; sensations of dryness appear to be mainly evoked by cold thermoreceptors. Mediators released by local inflammation, increase the excitability of eye polymodal nociceptors causing their sensitization and the augmented pain sensations. During chronic inflammation, additional, long-lasting changes in the expression and function of stimulus-transducing and voltage-sensitive ion channels develop, thereby altering polymodal terminal’s excitability and evoking chronic inflammatory pain. When trauma, infections, or metabolic processes directly damage eye nerve terminals, these display aberrant impulse firing due to an abnormal expression of transducing and excitability-modulating ion channels. This malfunction evokes ‘neuropathic pain’ which may also result from abnormal function of higher brain structures where ocular TG neurons project. Eye diseases or ocular surface surgery cause different levels of inflammation and/or nerve injury, which in turn activate sensory fibers of the eye in a variable degree. When inflammation dominates (allergic or actinic kerato-conjunctivitis), polymodal nociceptors are primarily stimulated and sensitized, causing pain. In uncomplicated photorefractive surgery and moderate dry eye, cold thermoreceptors appear to be mainly affected, evoking predominant sensations of unpleasant dryness.

## Introduction

Pain has been defined as “an unpleasant sensory and emotional experience associated with actual or potential tissue damage, or described in terms of such damage” [1]. In the eye, diseases leading to impaired vision such as cataract, retinal detachment or degeneration, or open-angle glaucoma course without pain, in spite of the accompanying damage to important eye structures. Nevertheless, pain is a symptom in a variety of other ocular pathologies, in particular those affecting tissues of the anterior segment of the eyeball and the orbit. The growing number of eye surface manipulations in the clinics (surgery, contact lenses) and the frequent exposure to artificial environments (air conditioning, video display terminals, air pollutants) is renewing the interest of eye care professionals for a better understanding of ocular pain [2••].

The qualitative and quantitative components of the pain experience vary, reflecting the complexity of the underlying peripheral and central neural processes. Sensory-discriminative nervous mechanisms map the origins of damaging events (mechanical, chemical, thermal) along with their location, their intensity and temporal aspects of the experience. The affective-motivational neural mechanisms of pain produce the accompanying emotional distress, which may have different quality and intensity. They have a compelling arousing and directive nature, comprising reflex responses, and complex behavioral reactions. Processing of the sensory-discriminative and affective-motivational features of pain is performed along the brainstem and multiple upper brain structures. These are subjected to intrinsic control by cortical, subcortical, and midbrain modulatory networks. Hence, the pain experience constitutes a highly distributed and complex brain function [3, 4].

## Physiological or Nociceptive Pain

The most obvious origin of eye pain is acute local injury. External physical or chemical stimuli acting on the eye at intensities near or over the level required to cause cell damage, stimulate a specific set of peripheral sensory nerve fibers generically named nociceptors [5]. These produce a discharge of nerve impulses that travel to the brain, encoding the spatial, and temporal characteristics of the noxious stimuli [6]. This sensory information is processed at various levels of the neuroaxis, finally reaching different areas of the cerebral cortex where it evokes pain sensations and unpleasant feelings referred to the eye, which persist for a variable period of time until healing takes place [7]. Such schematic description corresponds to the so-called “normal, physiological or nociceptive pain” an evolutionarily old mechanism aimed at protecting tissues from immediate potential or actual damage and promoting healing mechanisms, immobility, and rest [8]. Acute pain additionally triggers a number of stereotyped defensive responses that in the case of the eye includes reflex blinking and tearing, protective motor behavior (eye closure, head withdrawal, rubbing of the eye), and verbal expressive behavior [2••]. Hence, nociceptive pain alerts the organism of potential injury and due to its intrinsic and aversive nature, constitutes a well-defined, ancestral protection mechanism.

### Peripheral Origin

Nociceptors are peripheral sensory fibers acting as specific detectors for injurious stimuli [6]. They do not constitute a morphologically and functionally homogeneous population [9, 10]. In the eyeball, a fraction of them respond exclusively to noxious mechanical forces and are called mechano-nociceptors [11••]. The majority is also excited by mechanical stimuli and additionally respond to heat (>40 °C) and to a large variety of exogenous irritant chemicals, bacterial toxins, and endogenous inflammatory substances, being appropriately named polymodal nociceptors [11••, 12••, 13••]. Unlike the skin and non-keratinized surface mucosae, the eye surface does not receive the rich variety of low-threshold mechanoreceptor fibers that sustain cutaneous tactile sensitivity, although some low-threshold mechanoreceptors have been incidentally reported in the conjunctiva [14] and they are abundant in the lids, including palpebral conjunctiva. However, the eye surface is innervated by cold thermoreceptor fibers that extend into the cornea, limbus, and bulbar conjunctiva, and are sensitive to temperature drops [13••]. The majority of these cold thermoreceptor fibers are functionally similar to the canonical low-threshold cold thermoreceptors of the skin and the mucosae of mouth, tongue, and nose [15]; they exhibit a continuous impulse activity at the background tissue temperature, which increases prominently with small temperature drops [13••, 16, 17]. A reduced subset of cold-sensitive fibers does not exhibit spontaneous activity at background temperature and fire nerve impulses only when strong cooling is applied to the corneal surface [13••, 18]. Conceivably, these high-threshold cold receptors can be functionally classified as a particular subtype of nociceptors [19]. Each functional class of eye sensory nerve fiber evokes a particular modality of conscious sensation [20••, 21•].

### Central Representation

The cell somata of the sensory neurons innervating the eye surface, which are located in the TG, are heterogeneous not only in the above-described transducing properties of the endings, but also in size, molecular signature, and active and passive membrane properties [22, 23]. Their central axons terminate in two regions of the trigeminal brainstem complex, the trigeminal subnucleus interpolaris/caudalis transition region (Vi/Vc) and the caudalis/upper cervical cord junction (Vc/C1) [24•, 25] establishing direct or interneuron-mediated contacts with second-order projection ocular neurons [26••, 27••]. These, in turn, project preferentially to the parabrachial nucleus [28] and also to the posterior thalamus [29] and the insular cortex to convey information associated to the sensory-discriminative aspects of pain, and to the periaqueductal gray, hypothalamus, amygdala, and prefrontal cortex for the emotional aspects of pain [30]. Axons of second-order eye neurons primarily located in Vi/Vc, project to other brain areas involved in lacrimation (superior salivatory nucleus) and eyeblink (facial motor nucleus).

### Modulation

Strong physical or chemical actions on eye surface tissues destroy in a variable degree corneal and/or conjunctival cells. Inflammation results of the local release of a large variety of chemicals by injured local cells and by resident or migrating immune cells (epithelium cells, fibroblasts, mast cells, neutrophils, monocytes, platelets) [9]. Released chemical agents include among others H^+^, ATP and adenosine, protons, prostaglandins and leukotrienes, bradykinin, 5HT, platelet-activating factor, histamine, as well as cytokines such as interleukins and tumor necrosis factor and neurotrophins, like NGF [2••]. In addition, directly stimulated nociceptor nerve terminals release locally neuropeptides (Substance P, CGRP, Neurokinin A) [31]. Distant intact branches of the same parent axon become also depolarized by antidromic propagation of nerve impulses, thereby extending neuropeptide release to intact tissue areas. Neuropeptides further potentiate the liberation of mediators by inflammatory cells [32]. Inflammatory mediators evoke spontaneous impulse activity in nociceptor terminals, lowering of the stimulus threshold, and augmenting nerve impulse discharge generated by suprathreshold stimulation [9, 33]. These changes are jointly named ‘peripheral sensitization’ [34, 35]. Sensitization leads to spontaneous pain to allodynia, i.e., pain evoked by mechanical or thermal stimuli of innocuous intensities and to hyperalgesia, as it is named the exaggerated pain caused by mild noxious stimuli in the primary area of inflammation [1]. Hence, peripheral nociceptor sensitization is responsible of the distinct quality and persistence of pain arising from inflamed tissues.

## Neuropathic Pain

In certain circumstances, pain is not the result of a peripheral insult to the innervated tissue, but is caused by a direct injury or functional disturbance of the neural elements involved in the detection and processing of nociceptive stimuli [36, 37]. These elements include the peripheral nerve terminals and axons of the nociceptive sensory neurons located in the dorsal root and cephalic sensory ganglia; they also comprise the higher-order neurons of the spinal cord, brain stem, thalamus, and various other subcortical and cortical structures in charge of receiving and processing the peripheral nociceptive input. The abnormal, unpleasant sensation caused by the disturbed functioning of any of the components of the brain pain matrix is called ‘neuropathic pain’ [1] and is typically evoked at the periphery by sensory nerve traumatisms, metabolic diseases as diabetes, and by the action of a large variety of chemical, toxic, or infectious agents causing damage to peripheral sensory neurons at any point of their trajectory (peripheral neuropathic pain); within the central nervous system, damage to the pain-processing neuronal groups composing the pain network by ischemia, hemorrhage, mechanical compression, infections, or degenerative processes may also lead to central neuropathic pain.

## Transduction Mechanisms in Eye Nociceptors

Sensory nerve terminals innervating the eye are peripheral axons of neurons located in the trigeminal ganglion. These manufacture in their cell body different ion channel-transducing proteins that are transported to the peripheral endings. Transducing channels open when stimulated by a specific form of energy, thereby causing depolarization and a nerve impulse discharge that conveys sensory information to the brain. The specific sensitivity of each neuronal class to a given physical or chemical energy change is determined, at the molecular level, by the distinct expression in each ocular TG neuron class of different ion channel-transducing proteins. In addition, TG neurons express voltage-gated sodium (Na_v_), potassium (K_v_) and calcium (Ca_v_) ion channels, and ligand-gated channels, like hyperpolarization-gated cyclic nucleotide (HCN) channels that contribute to shape the final neuronal excitability, and are critical to modulate the frequency and firing pattern of nerve impulses generated at the peripheral sensory terminals in the ocular tissues [38•].

With the use of cytochemical, biophysical, and pharmacological tools, several classes of transducing channels have been identified in the aforementioned functional types of eye TG neurons. Transduction of mechanical forces by corneal mechano-nociceptor and perhaps, also polymodal nociceptor neuron endings is possibly mediated by Piezo2, a mechanosensory channel recently identified in low-threshold somatic mechanoreceptor neurons [39••]. Polymodal neurons, preferentially those that contain neuropeptides, also express the heat and proton-sensitive TRPV1 channel, which is additionally the main final target for several of the signaling pathways activated by membrane receptor proteins for inflammatory mediators [40, 41••, 42•, 43]. Eye polymodal nociceptor TG neurons in particular of the non-peptidergic subtype, also express TRPA1, an ion channel opened by exogenous irritant chemicals, strong cold, and endogenous agents such as reactive oxygen species and lipid peroxidation products [44–46]. As occurs with TRPV1, TRPA1 is activated, or sensitized downstream of inflammatory PLC-coupled receptor pathways by pro-inflammatory exogenous and endogenous agents, thereby mediating inflammatory pain sensitization as TRPV1 also does. Hence, these two channel classes appear to be the main detectors of many different irritants, endogenous chemicals, and heat [47, 48]. ASICs channels, another family of ion channels highly sensitive to acid, have been also recently identified in corneal polymodal neurons [49].

For temperature detection, TRPM8 channels appear to be critical in the sensing of temperature decreases by ocular cold thermoreceptor fibers [50•, 51•, 52•, 53]. Genetic ablation of this channel in mice renders cold thermoreceptor endings of the cornea silent and irresponsive to cooling [41••]. TRPM8 activity is also influenced by discrete osmolality changes, so that small increases in osmolality augment by this mechanism cold thermoreceptor activity in the eye [54••, 55]. Finally, background potassium channels like the K_2P_ channels, which stay open at basal tissue temperature act also as thermosensor channels because they get closed by cooling, thereby inducing depolarization and nerve impulse firing in cold thermoreceptors [56•, 57•]. As a general rule, sensory receptor neurons tuned to respond to modalities other than cold, counteract the unspecific depolarizing effects of exposure to low temperatures through the expression of another particular type of cold-sensitive potassium channel (K_v_1) whose ion current named IK_D_ opposes depolarization, thereby making them insensitive to temperature reductions [58, 59]. Variable expression of this same ‘break’ K_v_1 channels serves in canonical cold thermoreceptor neurons to finely adjust their threshold; low-threshold cold thermoreceptors which express abundantly TRPM8 channels have a low expression of K_v_1 channels, whereas high-threshold cold thermoreceptors exhibiting lower levels of TRPM8, also have a higher expression of K_v_1 ‘breaking’ channels and of the temperature-insensitive, K_2P_ leak potassium channel TASK-3, altogether requiring more cooling to be activated [38•, 58, 60••, 61].

**Fig. 1:**
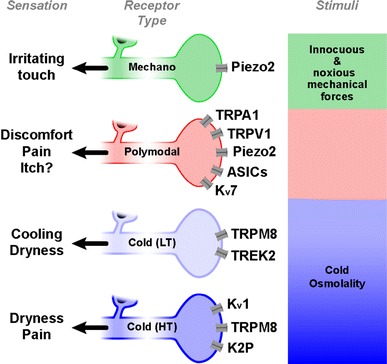
Schematic representation of the functional types of sensory neurons innervating the ocular surface and the main types of transducing channels expressed by their peripheral nerve terminals. The specific stimuli activating each neuronal class and the quality of sensations evoked by their activation in represented on the *right* side of the figure. The qualitative sensations attributed to each functional class of neuron is indicated on the *left* side. *LT* Low-threshold cold thermoreceptors, *HT* high-threshold cold thermoreceptors. Modified from: Belmonte C, Viana F. (Ref. [61])

Finally, a fraction of the TG neurons classified as nociceptors according to their neurochemical profile, contains low levels of TRPM8 channels, allowing them to fire in response to very strong cooling. In a part of these high-threshold thermosensitive neurons, expression of K_v_7.6 modulates the depolarization initiated by TRPM8 channels [62]. Figure 1 represents schematically the types of TG sensory neurons innervating the eye surface with their main transducing channels and the sensations possibly evoked by their activation.

## Pathobiological Modulation of Eye Sensory Fibers

Activity in sensory nerve fibers innervating the eye experience changes in various pathologies affecting the eye. Functional and morphological alterations of peripheral ocular fibers are due to inflammation and/or direct peripheral or central neuronal injury, two pathobiological processes accompanying eye diseases.

### Inflammation

Noxious stimuli not only activate directly nociceptors to evoke pain but in most cases, also cause tissue injury which leads to local inflammation. When inflammation occurs, the matching between the stimulus and the sensation of pain disappears. There is ongoing pain and tenderness of the tissue and the pain experienced under these circumstances is named ‘inflammatory pain.’

Inflammatory mediators locally released in eye tissues following injury or infection, diffuse to polymodal nociceptor nerve terminals where they interact with specific membrane receptor proteins (ligand-gated ion channels, G-protein-coupled receptors, cytokine receptors) [34]. The inflammatory agents open membrane ion channels, either acting directly on them or through activation of intracellular signaling cascades that phosphorylate and open TRPV1 and TRPA1 channels, causing membrane depolarization [63]. When this depolarization reaches the firing threshold, a discharge of propagated nerve impulses is generated; if the membrane potential remains below threshold, terminals become more excitable to subsequent stimuli, i.e., nociceptor endings are sensitized [64]. Notably, in intact cold thermoreceptors, inflammatory mediators such as bradykinin, prostaglandins, and histamine inhibit TRPM8 but do not do this through the conventional cell signaling pathways; the G-protein subunit Gqα instead binds to TRPM8 and when activated by a Gq-coupled receptor, directly inhibits ion channel activity [65••]. As a consequence, ongoing and cold-evoked impulse activity in cold thermoreceptors of the eye surface is reduced.

Inflammatory agents released by injury have other more permanent effects. When they activate intracellular kinases via their cognate receptors on the nociceptor terminals, they also produce posttranslational changes in transducer and voltage-gated ion channels. Consequently, the type, number, and distribution on ion channels manufactured by the neuron are modified. For instance, PKA activators promote the trafficking and increased insertion of Na_v_1.8 and Na_v_1.9 sodium channels into the plasma membrane, leading to altered thresholds and disturbed dynamic properties of the nerve membrane. Changes in the expression of potassium (K_v_), calcium (Ca_v_), and hyperpolarizing cyclic nucleotide-gated channels (HCN) further contribute to alter nociceptor nerve endings excitability [66, 67•, 68, 69]. This modified ion channel expression consolidates the spontaneous activity which underlies persisting pain sensations and the allodynia and hyperalgesia accompanying chronic inflammation.

The augmented nociceptor activity arising at peripheral sensory eye neurons of the TG travels centripetally to the trigeminal complex nuclei in the upper cervical spinal cord–brain stem area. This sustained nociceptor input not only generate pain, but also a phenomenon named ‘central sensitization’ [70]. This is defined as a long-lasting homo- and heterosynaptic facilitation of the transmission between central axonal branches of TG neurons and the projection neurons in the brainstem trigeminal complex, whereby the sustained input from nociceptor fibers enables subsequent facilitation of the response of these projection neurons [71–73]. Central sensitization is produced by nociceptors because these are the only peripheral fibers that co-release glutamate and neuropeptides. The combination of both neurotransmitters acting on postsynaptic glutamate receptors NMDA and AMPA, and on Substance P receptor NK1, determines the release within the projection neurons of sufficient intracellular calcium to increase postsynaptic membrane excitability [7]. Central sensitization causes secondary hyperalgesia and allodynia, thus contributing to enhance inflammatory pain.

### Nerve Injury

Lesion of peripheral nerve branches in the eye may occur directly, by physical damage, metabolic, toxic or viral neuropathy, or being part of an ocular tissue injury affecting also other non-neural local cell types and leading to inflammation.

When parent sensory axons and/or their terminal nerve branches are cut or destroyed, as occurs with surgical incisions performed for cataract, retinal detachment interventions, or photorefractive surgery, local inflammation is transient and of limited importance and nerve damage is the main adverse consequence of the trauma. The immediate, main functional effect of nerve division is the loss of sensitivity in the denervated territory, which may persist for days or months depending on the number of nerve branches affected and the location of the injury along the nerve trajectory (scleral nerve trunks, corneal stroma and sub-basal nerve branches, epithelial terminals) [74, 75••, 76•]. Transected axons rapidly seal off their central terminal stump, forming a terminal swelling from which fine branches (sprouts) begin to appear, growing rapidly into the denervated tissue to re-establish its innervation and tissue sensitivity [77•]. However, this process generally has a limited success and recovery of innervation density may be only partial; in the worst cases, blockade of growth of a fraction of the injured axons may lead to a tangled mass of aborted sprouts and end bulbs, forming microneuromas that may coexist with successfully regenerating fibers running in parallel [74]. Regeneration implies a very active structural and molecular reorganization of the injured eye neurons. Loss of nerve terminals impairs the capacity of the parent axons to transduce the stimulating energy, thereby decreasing their sensibility to natural stimuli; on the other hand, receptor molecules and ion channels follow the anterograde axonal transport to the sprouts of the regenerating nerve fibers, wherein they accumulate and are incorporated into the cell membrane. Nerve damage favors altered expression, posttranslational modification, and trafficking of transducing channels like TRPV1, TRPA1, or TRPM8 [78–80], of Na_v_, Ca_v_, and HCN channels, while the expression of background K_2P_ and K_v_ channels is down-regulated [66,  67•, 68, 69]. The drastic changes in expression, distribution, and phosphorylation of many ion channels in sensory neurons lead to modification of the intrinsic membrane properties, depolarization and generation of membrane potential oscillations resulting in abnormal nerve impulse bursts in the absence of a stimulus. This aberrant nerve activity is named ‘ectopic’ because instead of originating at the transducing terminals, it appears at the peripheral microneuromas that become abnormally sensitive to mechanical and chemical stimuli, including pH changes. These phenomena have been demonstrated in nociceptor fibers of various animal models of peripheral nerve injury [36, 81]. Changes derived from peripheral nerve injury are not limited to the affected nerve axons. Certain neuropathic pain states that result from peripheral nerve injury are accompanied by abnormal hyperexcitability of neurons within sensory ganglia [82], mediated by a glial reaction and local inflammation that give rise to cross excitation among neighboring TG sensory neurons [83].

The altered afferent input arriving from injured eye nociceptor neurons reaches the projection neurons of the trigeminal nuclear complex and produces central sensitization and enhanced pain [84]. When this central sensitization is caused only by an enhanced incoming nociceptor activity, healing reverses the phenomenon. However, after nerve injury, myelinated low-threshold mechanoreceptor and cold thermoreceptors fibers traveling in the injured nerve may undergo phenotypic changes, including increased expression of neuropeptides. They thus acquire the capacity to trigger or maintain central sensitization by acting on the nociceptor projection neurons that receive also an input from low-threshold receptors, thereby perpetuating pain and dysesthesias evoked by innocuous mechanical and thermal stimuli. Again, such spontaneous and abnormally triggered pain can paradoxically coexist with a reduced sensibility to natural stimuli due to the disturbed transducing capacity of the peripheral endings of injured eye nerve fibers [85•]. Altered neuronal excitability may be moderate and transient, ceasing when nerve regeneration is complete. However, in some cases, the distorted neuronal excitability persists after apparent healing of the injured tissue and neuropathic pain becomes a permanent problem [85•, 86].

## Peripheral Pain Mechanisms in Kerato-Conjunctivitis, Dry Eye Disease, Post-photorefractive Surgery, and Contact Lens Wearing

Ocular surface pathologies are the most common source of eye discomfort and pain. Other pathological processes involving inflammation of the uveal tract, like uveitis, acute congestive glaucoma, retinitis, or endophthalmitis also course with intense pain. The contribution of the different functional types of ocular sensory nerve fibers to pain in the posterior segment of the eye has not been defined in detail, although polymodal nociceptors seem to play a leading role in pain evoked by uveal stimulation [87, 88].

**Fig. 2:**
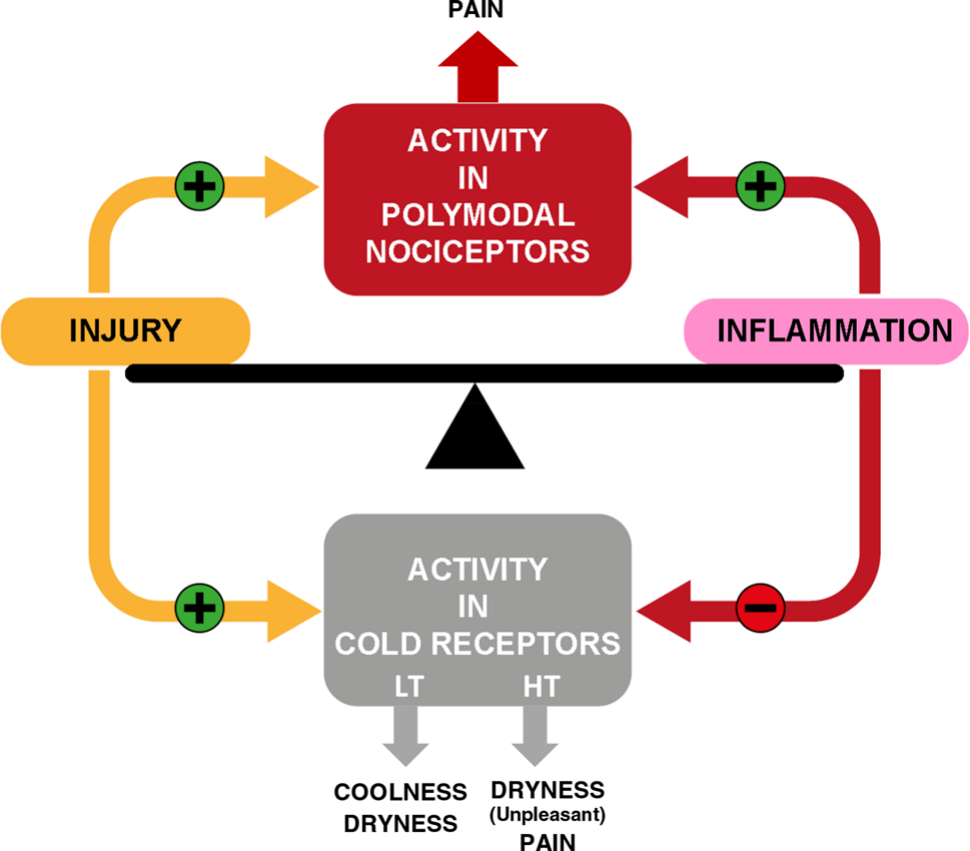
Schematic representation of the hypothetical influence of injury and inflammation on sensory terminals of TG neurons innervating the ocular surface. Inflammation activates directly and/or sensitizes polymodal nociceptor fibers, causing inflammatory pain while if these fibers are injured, they produce an abnormal, ectopic ongoing activity evoking neuropathic pain. Nerve injury induces on low-threshold cold thermoreceptors (LT) an abnormally high basal ongoing activity that elicits sensations of dryness with a cooling component; when high-threshold cold thermoreceptors (HT) become spontaneously active, unpleasant or painful dryness sensations are evoked. Contrarily, inflammation alone tends to silence TRPM8-dependent impulse activity in both subtypes of cold thermoreceptors

Pain and discomfort accompanying ocular surface pathologies arise from a variable combination of inflammation and nerve damage (Fig. 2), which influence mutually and change in each particular pathological condition [89]. Disturbances in the architecture and molecular signature of peripheral sensory nerves, TG somata, and synaptic connections of the brain projection neurons are thus different depending on the type of ocular surface disease. This explains the variable alteration of the impulse activity at the sensory nerve pathways in each case and thereby, the quantitative and qualitative differences between unpleasant eye sensations experienced in each ocular surface pathology.

Allergic conjunctivitis is primarily characterized by tissue inflammation accompanied by itching, grittiness, and burning of the eyes [90]. Recording of the various classes of corneal nerve terminals in an experimentally induced guinea pig model of allergic conjunctivitis [91] showed a reduced threshold of mechano- and polymodal nociceptors (i.e., these fibers responded to lower stimulus intensities); moreover, impulse response of polymodal nociceptors to noxious chemical stimuli was enhanced, altogether reflecting sensitization. In contrast, low-threshold cold thermoreceptor activity was depressed as it could be expected from the inhibition of TRPM8 by inflammatory mediators [65••]. Collectively, the overall changes in the firing response of corneal sensory fibers correlate well with the foreign body and itching sensations reported by allergic kerato-conjunctivitis patients. A similar, altered pattern of activity in nociceptors and cold thermoreceptors innervating the guinea pig eye surface was obtained after induction of an experimental, actinic kerato-conjunctivitis [92] which corresponds well with the subjective discomfort feelings reported by these patients.

Unpleasant dryness sensations are possibly the most prominent symptom in dry eye disease (DED) [93]. The initial physical stimuli for ocular surface receptors in aqueous-deficient and evaporative dry eye are evaporation-induced enhanced cooling and augmented osmolality, two potent activators of cold thermoreceptors [41••, 54••]. The scenario complicates later, with the apparition of inflammation and nerve damage resulting of the sustained dryness-induced injury of eye surface epithelium cells [94, 95] which affects polymodal nociceptors. In an experimental model of chronic dry eye produced by removal of the main lachrymal gland in guinea pigs [96], the discrete inflammation developed during the 1st week following surgery caused a moderate and transient sensitization of polymodal nociceptors. When inflammation subsided, polymodal nociceptors recovered in a great extent their normal behavior as occurs with mechano-nociceptors. In contrast, along the 1st month after removal of the lacrimal gland, guinea pig and mice cold thermoreceptors increase gradually their ongoing activity, in parallel with a progressive alteration of the morphological appearance of sensory fibers of the cornea, suggestive of nerve damage [96, 97••]. This enhanced ongoing activity is due to an augmented expression in corneal cold thermoreceptor neurons of N_av_ channels together with a reduced expression of K_v_ channels, which jointly induce a net increase in neuronal excitability [96]. The similarity of the functional changes seen in ocular cold thermoreceptor neurons during dry eye, with those caused by oxaliplatin, a neurotoxic chemotherapy agent which provokes neuropathy of cutaneous nerves and cold allodynia [98], further suggests that nerve injury is, at least partly behind the functional disturbances seen in cold thermoreceptors following chronic eye dryness, additionally enhanced by the stimulating effects of tear hyperosmolality [54••]. Such augmented activity could evoke the characteristic unpleasant sensations typical of DED, whose conscious ‘dryness’ quality resembles the feelings experienced in normal life when the intact eye surface is desiccated by strong air currents or low environmental humidity [99]. In more severe forms of DED, local corneal surface inflammation potentiated by the presence of inflammatory mediators in secreted tears [95, 100•, 101], expectedly activates also polymodal nociceptors, evoking additional sensations of burning pain.

Photorefractive surgery is often accompanied by discomfort sensations described as ‘eye dryness’ in spite of the absence in most of these patients of a reduced tearing [102•]. In the different surgical procedures employed for this surgical treatment, corneal sensory nerve endings are injured in a variable degree [103]. Damage impairs the transducing mechanisms and accordingly, polymodal and mechanosensory fibers innervating the injured area respond less to natural stimuli [104•]. In contrast, part of the cold thermoreceptors around the wounded area exhibits an abnormally augmented background activity and warmer thresholds for cooling. The damaging effects on polymodal and mechanonociceptor fibers explain the long-lasting reduction of mechanical and chemical sensitivity observed in post- photorefractive surgery patients [75••], whereas the presence of a ‘neuropathic’ activity in the injured corneal fibers would be the reason that discomfort after photorefractive surgery is qualitatively described as ‘dryness’ [105••].

**Fig. 3:**
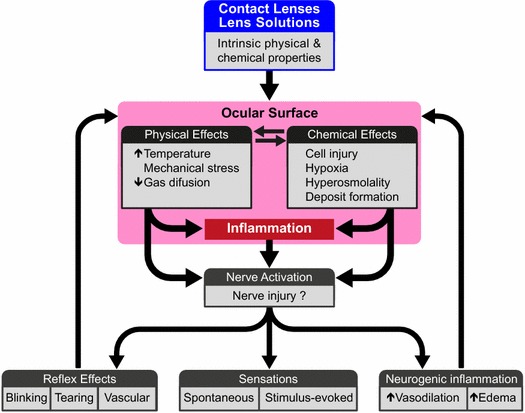
Hypothetical effects of contact lenses and eye lens solutions on ocular and lid surface tissues. Mechanical forces, temperature changes and chemical stimulation by exogenous irritants or release of endogenous agents consecutive to cell injury, hypoxia or pH and osmolality changes, will lead to sensory nerve stimulation, damage of nerve terminals and local inflammation. Local inflammation will further activate and sensitize sensory nerve fibers. These will evoke discomfort and pain, reflex effects and neurogenic inflammation

The influence of contact lens wearing on ocular surface sensations and the psychophysical attributes of discomfort sensations accompanying contact lens wearing has been extensively studied in humans [106••]. Nevertheless, direct knowledge of how contact lenses act on the peripheral and central neural mechanisms underlying sensations arising from eye tissues is lacking. Hypothetically, contact lenses and solutions used for rinsing and disinfection may act as mechanical and chemical stimulating forces for the cornea, bulbar conjunctiva, palpebral conjunctiva, and eyelid borders, thus becoming potential activators of their sensory nerve terminals. Lens wearing may additionally evoke with time, variable levels of inflammation, and/or nerve terminal injury in these tissues (Fig. 3). Direct stimulation of sensory nerve fibers by the contact lens is expected to activate a fraction of the various functional populations of sensory nerve fibers innervating the eyeball and lids, including nociceptors. Their activity will change with the development of local inflammation around nerve terminals and cause eventual nerve damage, as occurs in other ocular surface aggressions, leading to augmented discomfort and pain and reflex effects as vasodilation, increased tearing and blinking. Inflammation can be amplified further through neuropeptide release by the activated peptidergic nerve terminals [106••]. However, in contrast with the knowledge obtained for photorefractive surgery or eye dryness, no experimental data are available about the type of nerve fibers activated by the lens in the different eye tissues, the temporal evolution of their activity with continued lens use and the contribution of inflammation and nerve injury to impulse activity in the stimulated functional types of sensory receptors. The absence of information on these questions is surprising, considering its potential importance for the design of new lenses and materials aimed at alleviating discomfort caused by contact lens wearing and deserves experimental attention. Figure 3 represents schematically the hypothetical actions of contact lenses and contact lens solutions on sensory nerve fibers of the ocular surface, which would lead to nerve activation and the corresponding sensory, reflex and inflammatory responses.
